# Supplementary material for: KRAS Copy Number Gain in Cell-Free DNA Analysis-Based Liquid Biopsy of Plasma and Bile in Patients with Various Pancreatic Neoplasms
Source: Int J Mol Sci. 2025 Sep 9;26(18):8763. doi: 10.3390/ijms26188763 (PMC12469631; doi:10.3390/ijms26188763)
Supplement: Supplementary file 1 [file ijms-26-08763-s001.zip › Figure S3_Jain_et_al._2025.pdf]

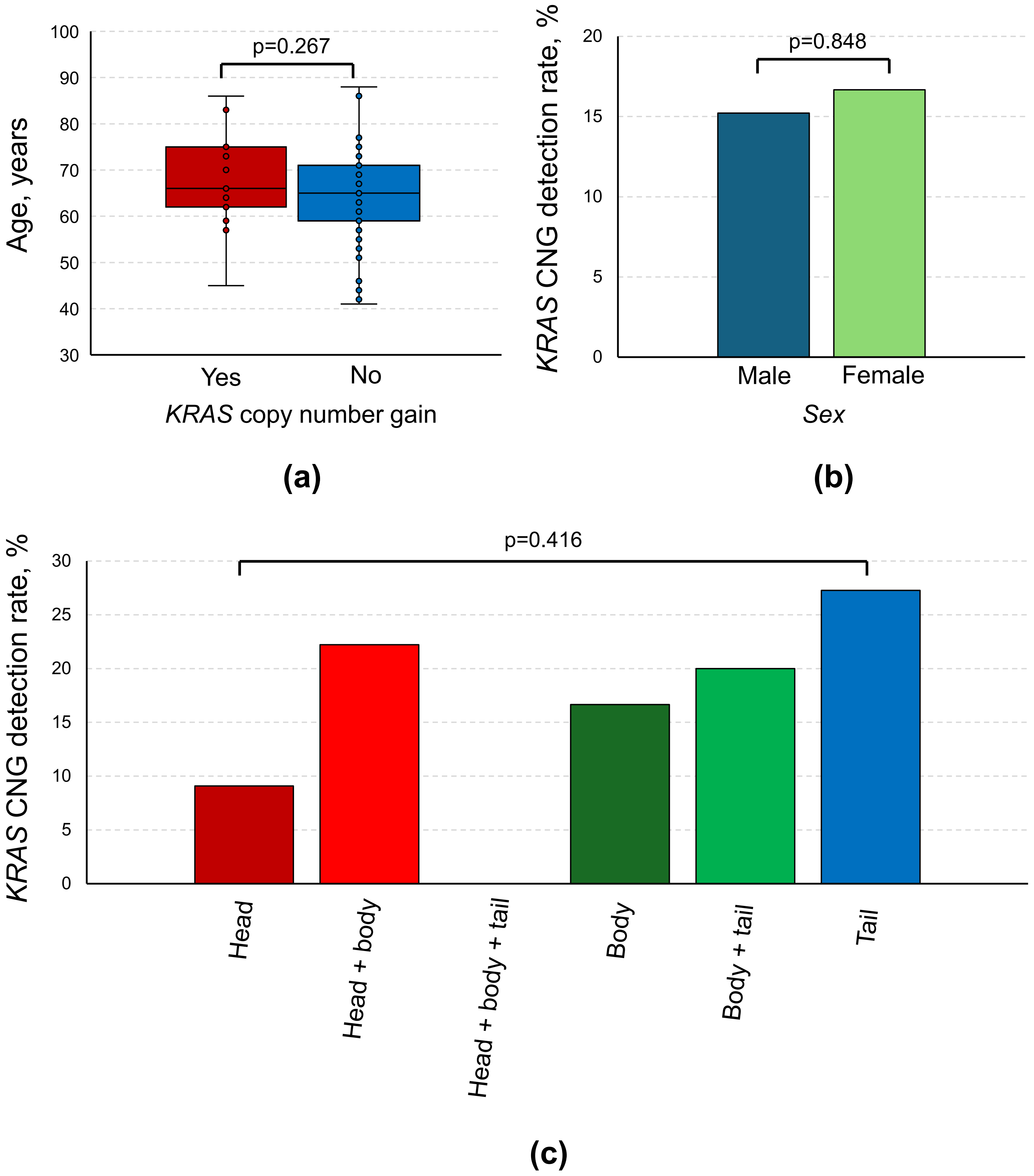

p=0.267
Age, years
100
90
80
70
60
50
40
30
Yes
No
KRAS copy number gain
(a)
p=0.848
KRAS CNG detection rate, %
20
15
10
5
0
Male
Female
Sex
(b)
p=0.416
KRAS CNG detection rate, %
30
25
20
15
10
5
0
Head
Head + body
Head + body + tail
Body
Body + tail
Tail
(c)

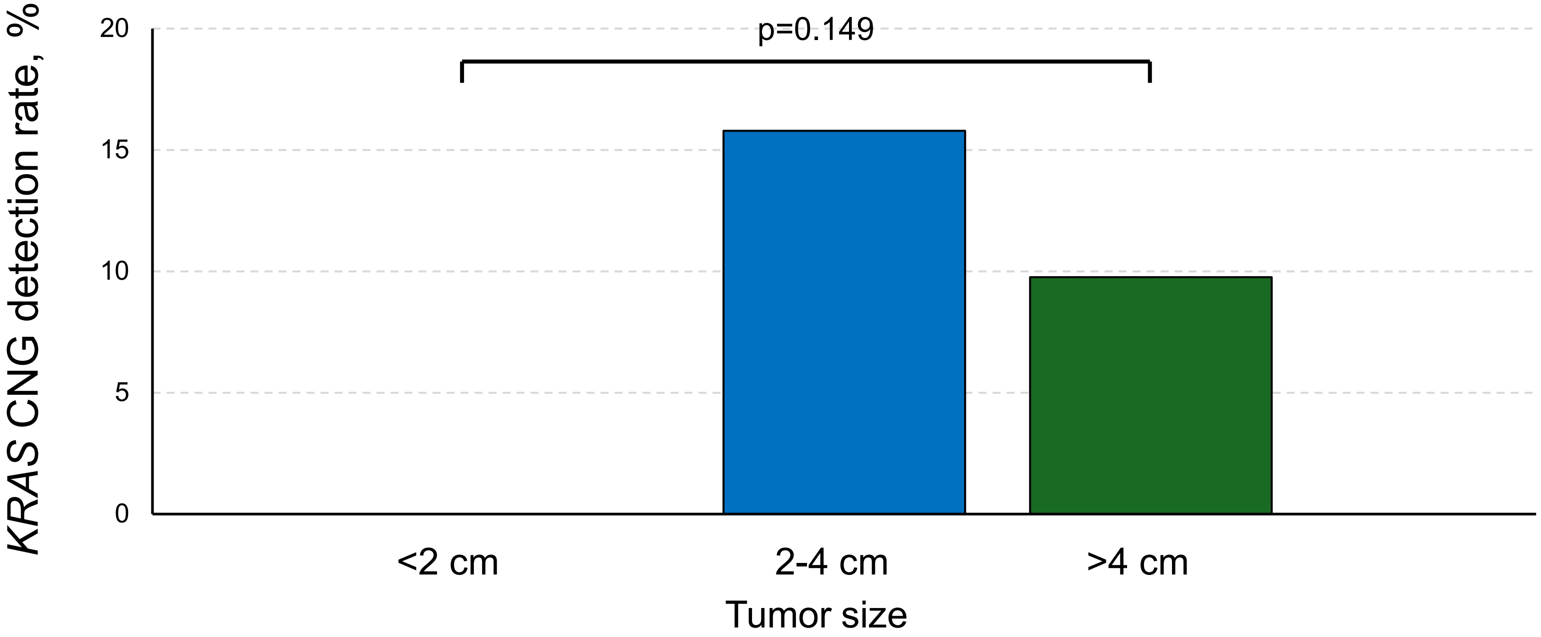
KRAS CNG detection rate, %
20
15
10
5
0
p=0.149
<2 cm
2-4 cm
>4 cm
Tumor size

(d)

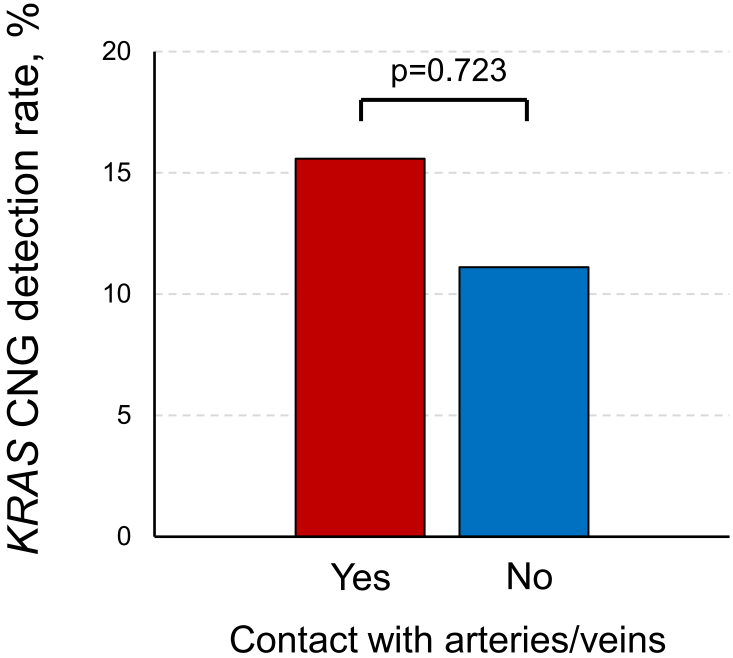
KRAS CNG detection rate, %
20
15
10
5
0
p=0.723
Yes
No
Contact with arteries/veins

(e)

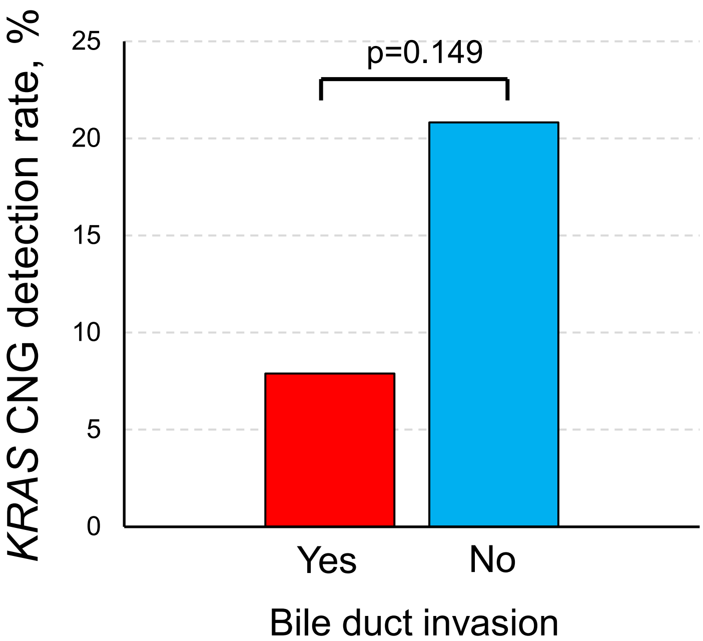
KRAS CNG detection rate, %
25
20
15
10
5
0
p=0.149
Yes
No
Bile duct invasion

(f)

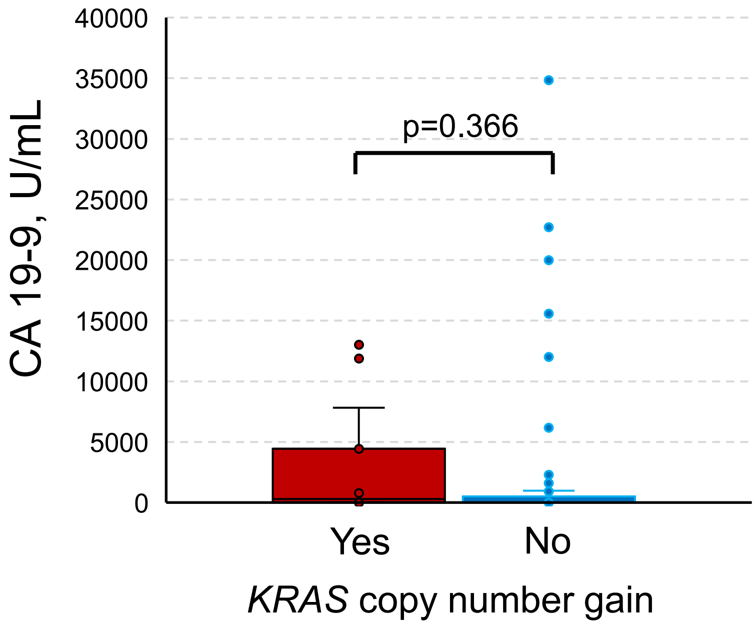

(g)

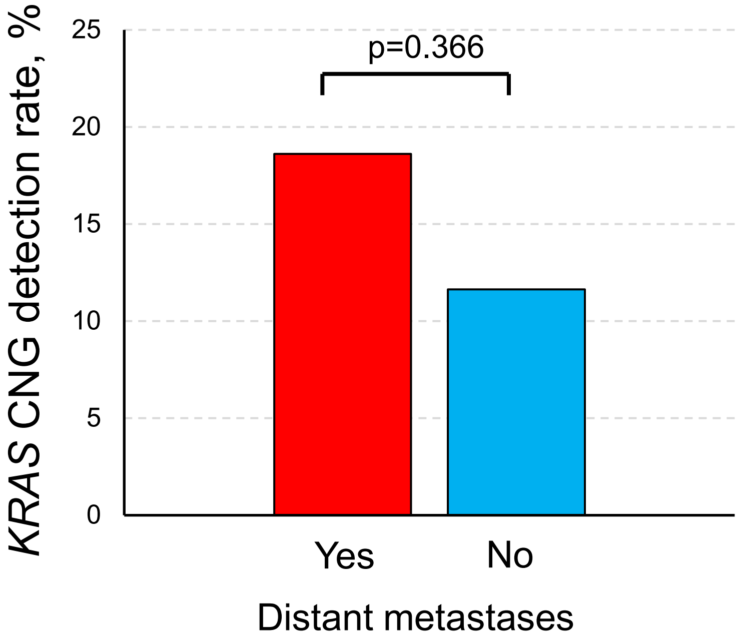

(h)

Figure S3. Association of *KRAS* CNG analysis results in plasma cell-free DNA with demographic and clinical data. (a) Age. (b) Sex. (c) Tumor localization. (d) Tumor size. (e) Contact with arteries/veins. (f) Bile duct invasion. (g) Serum CA 19-9 levels. (h) Distant metastases. CNG, copy number gain. Data for bile is not presented due to low sample size for CNG-positive cases.
