# Supplementary material for: KRAS Copy Number Gain in Cell-Free DNA Analysis-Based Liquid Biopsy of Plasma and Bile in Patients with Various Pancreatic Neoplasms
Source: Int J Mol Sci. 2025 Sep 9;26(18):8763. doi: 10.3390/ijms26188763 (PMC12469631; doi:10.3390/ijms26188763)
Supplement: Supplementary file 1 [file ijms-26-08763-s001.zip › Figure S1__Jain_et_al._2025.pdf]

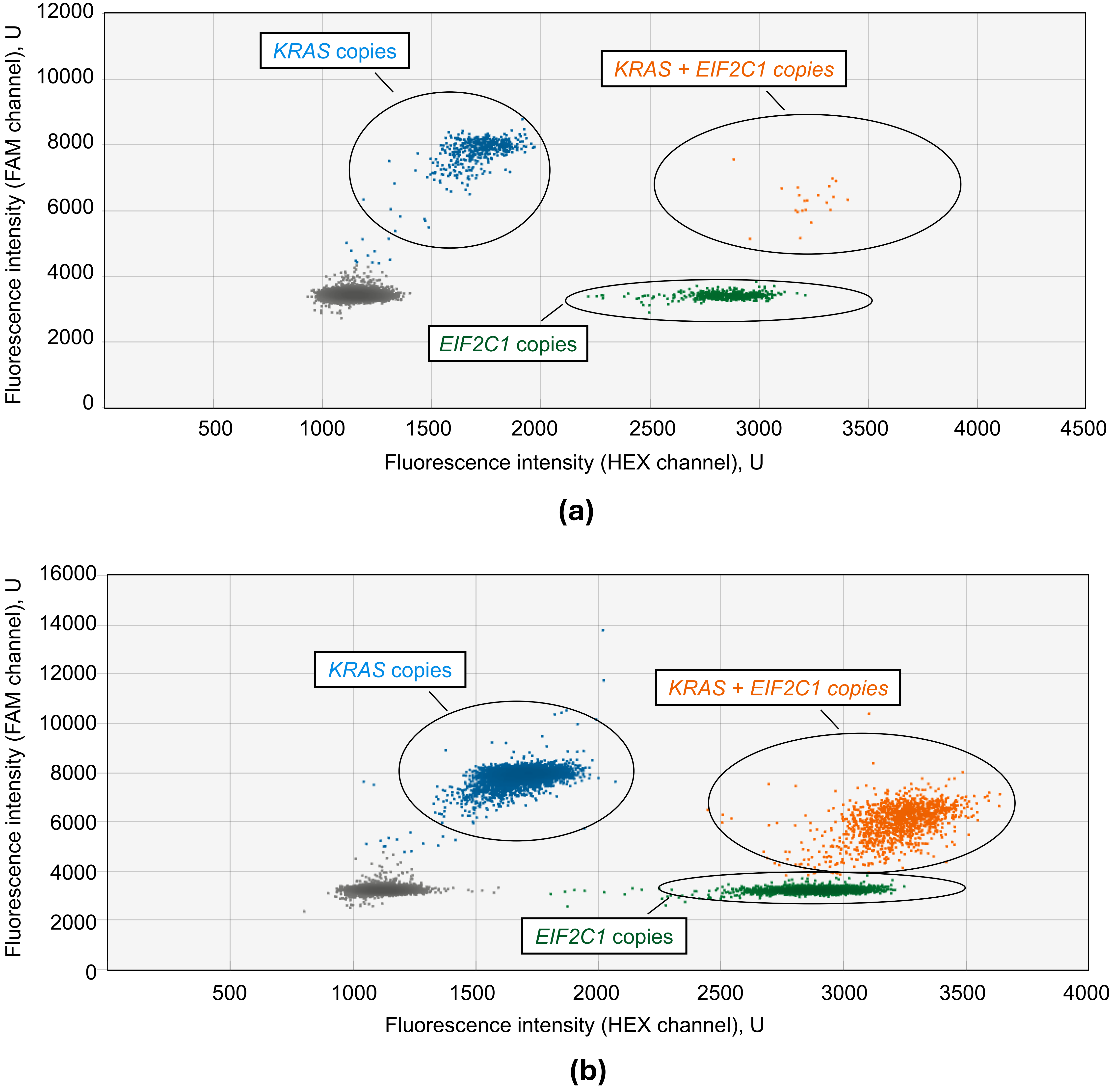

Figure S1. Examples of 2D diagrams generated during ddPCR using the developed *KRAS* CNG detection assay. **(a)** Plasma sample. **(b)** Bile sample. Each dot on the diagrams represents a single emulsion droplet. Detailed description of the developed assay is available in Table 2.
