# Supplementary material for: KRAS Copy Number Gain in Cell-Free DNA Analysis-Based Liquid Biopsy of Plasma and Bile in Patients with Various Pancreatic Neoplasms
Source: Int J Mol Sci. 2025 Sep 9;26(18):8763. doi: 10.3390/ijms26188763 (PMC12469631; doi:10.3390/ijms26188763)
Supplement: Supplementary file 1 [file ijms-26-08763-s001.zip › Figure S2_Jain_et_al._2025.pdf]

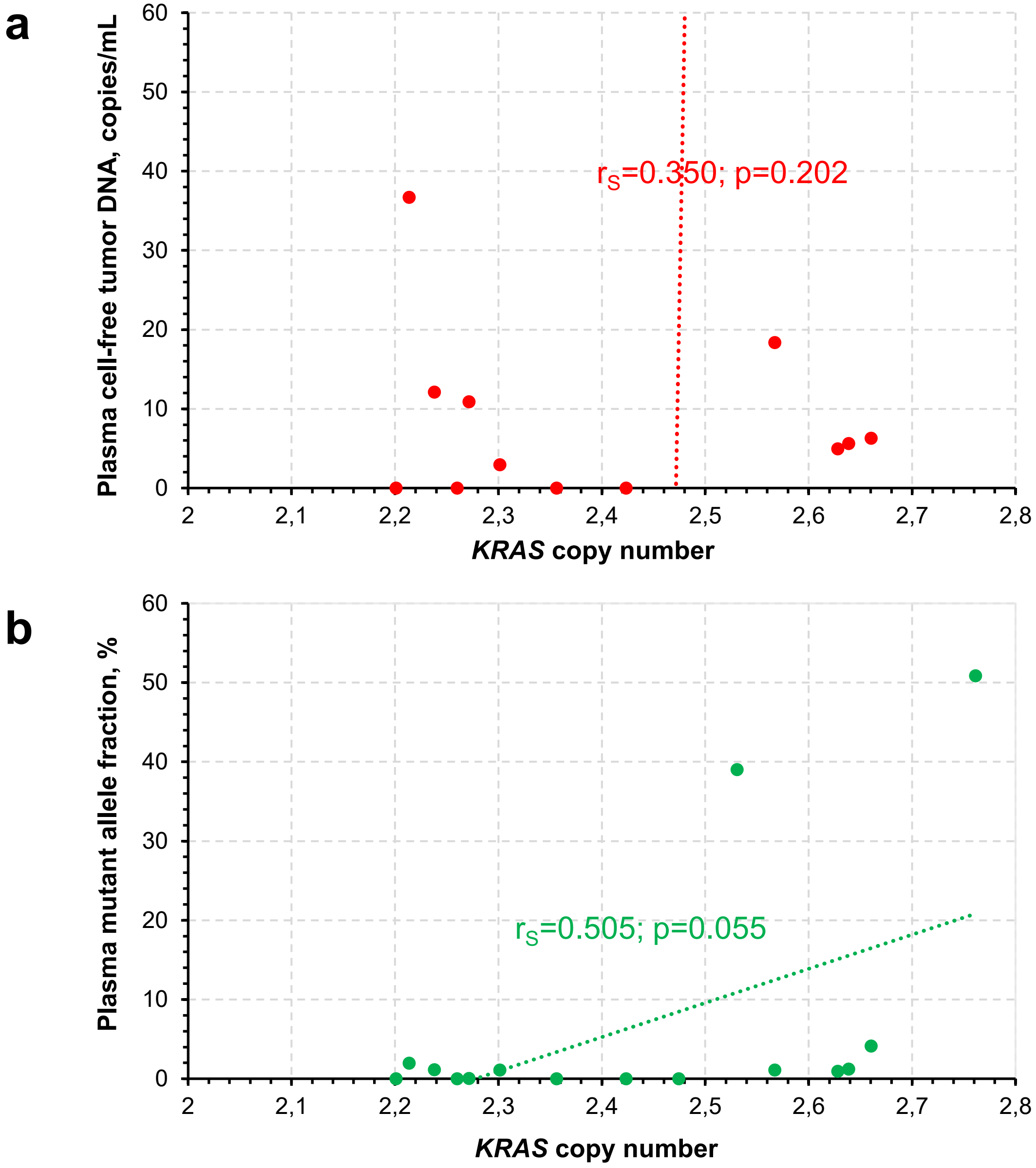

Figure S2. Correlation of circulating tumor DNA carrying common *KRAS* mutations in G12, G13, Q61 hotspots with *KRAS* copy number in the *KRAS* CNG positive plasma samples of patients with pancreatic ductal adenocarcinoma. **(a)** Scatter plot for cell-free tumor DNA concentration (copies per 1 mL of biomaterial) in plasma (outliers with cell-free tumor DNA concentration of more than 3000 copies/mL were excluded to preserve the proportions of the plot). **(b)** Scatter plot for mutant allele fraction in plasma. CNG – copy number gain; r_S – Spearman's rank correlation coefficient.
